# State policy influence on the early diffusion of buprenorphine in community treatment programs

**DOI:** 10.1186/1747-597X-3-17

**Published:** 2008-06-20

**Authors:** Lori J Ducharme, Amanda J Abraham

**Affiliations:** 1 Institute for Behavioral Research, 111 Barrow Hall, University of Georgia, Athens, GA 30602, USA

## Abstract

**Background:**

Buprenorphine was approved for use in the treatment of opioid dependence in 2002, but its diffusion into everyday clinical practice in community-based treatment programs has been slow. This study examines the net impact of efforts by state agencies, including provision of Medicaid coverage, on program-level adoption of buprenorphine as of 2006.

**Methods:**

Interviews were conducted with key informants in 49 of the 50 state agencies with oversight responsibility for addiction treatment services. Information from these interviews was integrated with organizational data from the 2006 National Survey of Substance Abuse Treatment Services. A multivariate logistic regression model was estimated to identify the effects of state efforts to promote the use of this medication, net of a host of organizational characteristics.

**Results:**

The availability of Medicaid coverage for buprenorphine was a significant predictor of its adoption by treatment organizations.

**Conclusion:**

Inclusion of buprenorphine on state Medicaid formularies appears to be a key element in ensuring that patients have access to this state-of-the-art treatment option. Other potential barriers to the diffusion of buprenorphine require identification, and the value of additional state-level policies to promote its use should be evaluated.

## Background

The treatment of opioid addiction in the U.S. is undergoing significant change. In October 2002, the Food and Drug Administration (FDA) approved buprenorphine (Subutex^® ^and Suboxone^®^) for use in the treatment of opioid dependence. Interim rules promulgated pursuant to the Drug Addiction Treatment Act (DATA) of 2000 opened two distinct delivery systems for buprenorphine. First, and uniquely, primary care physicians (with appropriate training and certification) are permitted to prescribe buprenorphine in office-based settings, provided that adequate linkages to counseling and other support services are made available. Second, opioid treatment programs (OTPs, once referred to as methadone maintenance programs) are permitted to dispense buprenorphine, but under restrictions that parallel the manner in which they presently dispense methadone. Somewhere in the middle are traditional community-based treatment programs (often somewhat inelegantly described as "drug free" modalities), which either can offer buprenorphine through an approved staff physician, or may affiliate with office-based physicians to provide counseling and other support services for patients receiving buprenorphine in primary care settings.

It is generally hoped that the confluence of these changes will significantly benefit patients in need of opioid addiction treatment by increasing the number and availability of treatment options. However, these recent changes at the Federal level are necessarily mediated by State-level regulations and policies, which may affect the rate of adoption of buprenorphine, particularly in OTPs and community-based treatment settings. Each state has its own regulations for addiction treatment, including licensure and certification, financing (including disbursement of block grant funds, and setting Medicaid formularies and coverage limitations), and other domains that may influence programs' ability to adopt buprenorphine (for example, rules for program licensure and use of medications). State regulations may create or remove barriers to the adoption of buprenorphine by community-based treatment provider organizations. Understanding the role of the state regulatory context in the diffusion of buprenorphine can contribute to a broader understanding of the innovation diffusion and technology transfer processes.

The diffusion of buprenorphine exemplifies the recent emphasis on the adoption of evidence-based practices within the addiction treatment field. There is ongoing concern about the slow rate of adoption of treatment approaches that have been developed and validated in recent years [1,2]. Several studies have examined the process of technology transfer within single treatment units [3-5], while others have examined the perceptions and experiences of individual clinicians regarding specific new treatment methods [6-11]. Despite their importance, much less attention has been paid to the organizational characteristics associated with the adoption and implementation of treatment innovations [12,13]. Even less attention has been paid to the state regulatory and funding contexts which circumscribe treatment providers' service delivery options.

Generally, the management literature on innovations, particularly the work of Rogers [14], has been used to inform the development of hypotheses about the relationship between the characteristics of the innovation, the characteristics of the potential adopters, and the relationships between those characteristics and the likelihood of innovation adoption. In terms of the innovation, Rogers notes that its relative advantage, compatibility, complexity, trialability, and observability all influence the rate of adoption. Buprenorphine appears to have the "right mix" of these characteristics from the perspective of community treatment programs. To the extent that programs are already treating opioid-dependent clients, and/or are using medications in the treatment of addiction, buprenorphine is compatible with current practice. The medication is less "complex" than other pharmacotherapies (e.g., methadone) or even some psychosocial treatment techniques that require a significant investment in counselor training and clinical supervision. Buprenorphine presents a relative advantage over methadone, in that the legal, regulatory, and abuse and diversion issues are minimized. Trialability relates to the ability to "test" the drug on a limited basis before fully committing to its adoption. By establishing linkages with a DATA-waived physician, or obtaining a waiver for an existing staff physician, programs can "test" the use of buprenorphine without making a long-term commitment or expensive investment. Finally, the effects of buprenorphine are highly observable, and indeed in recent clinical trials program staff expressed a preference for it over clonidine, because the advantages in terms of its physiological effects on patients are especially noticeable [15].

Analyses of early organizational adopters of buprenorphine [16,17] identified numerous structural features of treatment programs as important predictors. Treatment programs that already had the necessary infrastructure in place to support the use of pharmacotherapies were significantly more likely to have adopted buprenorphine in its first 3 years of its availability. Buprenorphine was significantly more likely to be used in programs having one or more physicians on staff, based in hospitals, offering medically-managed detoxification services, treating proportionately more opioid-dependent patients, and having previously adopted one or more addiction pharmacotherapies. In addition, and net of these other factors, programs relying predominantly on self-paying and privately-insured patients were also significantly more likely to have adopted buprenorphine.

Organizational reliance on private insurance and other non-governmental funds has been a consistent predictor in analyses of medication adoption in addiction treatment, including alcohol pharmacotherapies such as naltrexone, disulfiram, and acamprosate [11,18-22], as well as psychotropic medications [23]. Such findings suggest that programs treating patients who can afford – or have insurance that will cover – medications are more likely to adopt and offer them. But substantial numbers of addiction treatment patients in the U.S. rely on public subsidies to pay for their care. For example, the Substance Abuse and Mental Health Services Administration [24] reports that 48% of treatment admissions in 2005 listed Medicaid or "other government sources" as their primary expected or actual form of payment. If state and local payers do not include buprenorphine as a covered benefit, many patients will be unable to afford the medication, and the providers from which they seek care may be less likely to offer it. The relationship between state-level policies and program-level service delivery offerings has not been systematically explored.

For the most part, the innovations literature assumes that would-be innovators make adoption decisions based on the characteristics of the innovation and the organization's capacity to integrate it into its operations. But issues such as patient payment methods point towards the broader contexts within which such decisions are made, and these contexts provide important incentives and barriers to the adoption of pharmacotherapies in addiction treatment. Institutional theory provides a framework for understanding the relationship between organizational decision-making and the broader operating environment [25]. An institutional perspective views organizations as dependent on their environment for both incentives to adopt innovations, as well as justifications to legitimate their actions [26,27]. Regulatory agencies (including accrediting bodies, payers, and state licensing bodies) set the tone for innovation by promoting policies and "model programs," and in the case of addiction treatment, define clinically acceptable standards of care. By defining policies and payment structures, states can be influential in encouraging or impeding the adoption of evidence-based practices at the organizational level. While institutional theory provides a conceptual basis for considering state policies in the adoption of medications, these remain understudied. This context is of specific concern in the case of buprenorphine.

### State Environments and Organizational Innovation: Making the Link

There is scant research that has focused on states as contexts for innovation, or as change agents in the innovation diffusion process. However, there is some research to suggest that attending to these environmental issues could enhance our understanding of the innovation process within organizations that are subject to state oversight. For example, Koyanagi et al. [28] reviewed state Medicaid coverage for psychiatric medications, and found substantial variation among states not only in the policies used, but in their application to different medications. Such variation might explain providers' decisions to incorporate these medications into their routine clinical practice.

Another study of this type [29] reviewed the availability of coverage for methadone maintenance in state Medicaid managed care programs in 1998. This study demonstrated substantial variation across states in terms of the nature and extent of benefits for persons seeking methadone treatment. Among the 50 states and the District of Columbia, they identified 25 states with no Medicaid coverage for methadone maintenance (including 8 states that, at the time, did not have any operating methadone programs); 13 states that excluded methadone from their Medicaid managed care plans; and 12 states where methadone services were included in Medicaid managed care plans, although service limits were imposed in 10 of those states. Although the opioid treatment system has substantially changed since this article was written, its findings provide some indication of the potential scope of variation that may be seen in the current state-level substance abuse funding environment, which may in turn be expected to influence adoption of new forms of treatment.

Research integrating state policy data and organizational- or patient-level data is rare in the behavioral healthcare literature, but a few examples suggest potential research directions. D'Aunno and colleagues have measured the influence of key external stakeholders on addiction treatment programs, finding associations between accreditation/licensure status and the adoption of HIV prevention practices [30] as well as the provision of methadone maintenance services meeting evidence-based standards for good clinical practice [31,32]. Such findings demonstrate that treatment providers may implement programming at least in part in response to the demands of agencies to which they are accountable. In other work, Goldman et al. [33] reviewed state Medicaid and other public assistance programs for HIV-positive patients, and concluded that patients' economic outcomes were predicted in part by the generosity of state coverage benefits for HIV/AIDS medications. While those analyses did not integrate organizational-level data on service provision and availability, they are suggestive of potential linkages between state policy and patient outcomes.

A recent study on the association between state-level factors and program-level adoption of naltrexone is especially informative [34]. While naltrexone has been available for the treatment of alcohol dependence since 1994, uptake among community treatment programs has been stagnant for quite some time. State-level factors including general Medicaid policies (e.g., limits on substance abuse treatment benefits, inclusion of generic drugs on the formulary), funding for public welfare, and population characteristics explained 16% of the variance in program-level adoption of naltrexone as of 2003. While the study controlled for a host of state-level factors, it did not specifically gather data on whether states' Medicaid programs provided coverage for the use of naltrexone as a treatment for alcohol dependence. Thus, while a sizeable proportion of the total variance in program-level naltrexone adoption was explained by characteristics of their states' policies, the direct relationship between availability of Medicaid coverage for a specific medication and program-level prescribing behavior remains unmeasured.

A final case study warrants mention, as it continues to provide important lessons for the opioid treatment field. That case involves the failed diffusion of levo-alpha-acetylmethadol (LAAM), a long-acting opioid agonist approved by the FDA in 1993. At the time of its introduction, LAAM appeared to offer several advantages relative to methadone, in particular the ability to maintain patients on a thrice-weekly (instead of daily) dosing schedule. Clinicians writing at the time of LAAM's approval expressed optimism about the potential for LAAM to increase access to treatment, integrate opioid addiction treatment into mainstream medicine, enhance providers' and patients' otherwise-limited treatment options, and achieve clinically advantageous results with less frequent dosing [e.g. [35,36]]. However, three years after its approval, few clinics were dispensing LAAM, and few inroads had been made in its diffusion [37]. For a number of reasons, production of the medication was ultimately discontinued by the manufacturer in 2003.

While some of the barriers to the diffusion of LAAM stemmed from resistance among the methadone treatment community itself, there was widespread consensus that layers of state and local regulatory barriers were as much to blame for its low rate of adoption [38]. State regulations for methadone needed to be modified to include LAAM, and rescheduling of the drug as a controlled substance was often required at the state level; in many cases, states failed to act on these measures [39]. As a result, states' inaction impeded the adoption of LAAM by OTPs. A growing consensus that the over-regulation of the opioid addiction treatment field was impeding the delivery of optimal clinical care led to the development of a new accreditation-based oversight system for the field. It is in this new context of federal oversight, and perhaps informed by lessons learned from LAAM, that buprenorphine was introduced.

This paper extends previous research on the predictors of buprenorphine adoption by examining structural, resource, and environmental variables in the U.S. population of substance abuse treatment programs. Controlling for organizational characteristics, the orientation and actions of state regulatory agencies are examined, and their net effect on program-level clinical decisions is estimated.

## Methods

### State Agency Interviews

In mid-2006, we conducted brief telephone interviews with at least one staff member in the agency having primary oversight responsibility for the use of buprenorphine in each state. Our initial point of contact in every state was the single state agency (SSA), which is responsible for oversight of addiction services and disbursement of block grant funds. In 29 states, we were directed to speak with the State Methadone Authority (SMA) for information about buprenorphine. Information was obtained from 49 states; one state did not respond to repeated requests for an interview. Respondents were asked a number of questions about the state's policies related to the use of buprenorphine for addiction treatment, including the availability of Medicaid coverage, the presence of any state requirements above and beyond prevailing Federal requirements for the prescription of the medication, and the general disposition of the state office toward the use of buprenorphine in community-based treatment programs. The responses to several key questions were utilized as predictor variables in multivariate models examining program-level adoption of buprenorphine.

State agency staff were asked whether buprenorphine was a covered Medicaid benefit for addiction treatment. Although we collected some detail on the nature and extent of coverage provided (e.g., special populations, pre-approval requirements, and prescription limits), there was insufficient variation in these responses to meaningfully differentiate states based on this detailed information, as are often used in analyses of other, more common, medications. Thus, for these analyses, we use a simple dichotomous variable measuring whether Medicaid provides any coverage for buprenorphine in the state. In all, 28 states reported having *Medicaid coverage for buprenorphine *at the time of our interviews in 2006. (These states were AK, AZ, CA, DE, FL, HI, IL, MD, MA, MI, MN, NE, NJ, NM, NY, NC, ND, OH, PA, RI, SD, TN, VT, VA, WA, WV, WI and WY.)

A series of additional questions measured the extent to which each of the responding state agencies encouraged or actively facilitated the adoption of buprenorphine by community treatment programs. First, we asked whether the state agency had distributed to treatment providers two recent SAMHSA-authored clinical guidelines on the use of medication-assisted therapy for opioid dependence [40,41]. In all, 23 of the responding agencies reported that they had distributed one or both of these publications either in hard copy or via internet links. Fifteen state agencies reported that they had not distributed these materials, while eleven state agency respondents reported being unfamiliar with these treatment improvement protocols.

The tenor of SSA/SMA guidance to OTPs regarding buprenorphine was also informative. There is variability among states in the extent to which buprenorphine is viewed as a preferable alternative to methadone, and whether mainstream treatment facilities are viewed as preferable loci of care to OTPs. Along these lines, 7 states had requirements that OTP advise their clients about the availability of buprenorphine; an additional 14 states actively encouraged OTPs to do so. The remaining states had provided no such guidance to OTPs. When asked a more general question about the state agency's orientation toward buprenorphine, 23 respondents characterized their state agency as "actively encouraging" the use of buprenorphine by addiction treatment providers; the remaining 26 indicated that their agency had taken "no real position" on buprenorphine. No respondents characterized their state as having a negative or discouraging position on this medication.

The responses to these questions were nearly perfectly correlated with reported distribution of the SAMHSA clinical guidelines; that is to say, states that actively encouraged the use of buprenorphine were also those that encouraged OTPs to inform clients about it, and distributed the SAMHSA treatment manuals. Thus, we reduced these three items to one measure for this analysis, namely, whether the state agency *actively encouraged the use of buprenorphine*.

All respondents were asked about any then-pending regulatory changes, in an effort to determine whether there was variability in the amount of activity surrounding this medication. Few states anticipated any forthcoming regulatory changes pertinent to the use of buprenorphine in community-based treatment settings. The few pending regulatory changes mentioned were procedural in nature, for example, clarifying payment and approval processes for prescribers and pharmacy boards. Due to lack of variation on these items, they are omitted from these analyses.

### Treatment Facility Data

To examine the impact of regulatory and funding policies on the adoption of buprenorphine, data from a broad variety of treatment programs across the U.S. is essential. To ensure adequate representation of treatment programs across states, the 2006 National Survey of Substance Abuse Treatment Services (N-SSATS) was used for analysis. The N-SSATS is an annual survey of the population of facilities offering addiction treatment services in the U.S., conducted by SAMHSA. Although the N-SSATS is limited in terms of potential predictor variables, it is nevertheless the closest approximation to a census of the nation's treatment providers. The public use data file for the 2006 survey was obtained from the Substance Abuse and Mental Health Data Archive [42]. The public use data file contains no facility identifiers; however, codes for state, county, and metropolitan area are included on each record.

The entire 2006 N-SSATS public use datafile includes 13,771 facility records. These records were filtered to exclude facilities that did not deliver substance abuse treatment services. Specifically, facilities were excluded if they exclusively provided intake, assessment and/or referral services, along with those focusing exclusively on the provision of services for persons arrested for driving under the influence. Because our interest was in the adoption of buprenorphine by organizations rather than individuals, solo practitioners were also excluded from these analyses. Finally, facilities operating in Puerto Rico and the other US territories were excluded, because no data were collected from their respective local government oversight agencies about relevant regulatory and funding policies for buprenorphine. These exclusions resulted in a final data set of 12,236 substance abuse treatment facilities.

### Measures

Several variables available on the N-SSATS file were included as control or predictor variables in examining patterns of buprenorphine adoption. These can be grouped into three broad categories: ownership, facility characteristics, and funding sources. *Ownership *is defined in terms of three mutually-exclusive dichotomous variables: government-owned, private non-profit, and private for-profit. Because studies have repeatedly documented higher rates of pharmacotherapy adoption in for-profit facilities, these are used as the reference category in the multivariate model.

Facility characteristics include several variables that may be associated with an organization's willingness or ability to adopt medications. First, because hospital-based programs have the infrastructure to support the use of pharmacotherapies, they may be more likely to adopt buprenorphine. Facilities self-reported whether they were *located in a hospital *(1 = yes, 0 = no). Next, because buprenorphine is indicated for the treatment of opioid addiction, *opioid treatment programs (OTPs) *may be more likely to adopt it. Programs are coded as OTPs if they self-reported this status to N-SSATS (1 = yes, 0 = no). Third, the N-SSATS includes organizations that may vary in the proportion of total activity devoted to addiction treatment, ranging from self-contained specialty addiction treatment programs and OTPs to units within general hospitals and mental health centers. Organizations having *substance abuse treatment as their central focus *are expected to be more likely to adopt addiction pharmacotherapies such as buprenorphine. This dichotomous variable is coded 1 if the facility's primary focus is substance abuse treatment, and 0 otherwise.

Because much of the early clinical research on buprenorphine has focused on its use in detoxification protocols, treatment programs *offering detox services *may be more inclined to adopt it. Programs self-reported whether they offered detoxification among their array of services (1 = yes, 0 = no). Buprenorphine provides community treatment programs an option for treating opioid-dependent clients without investing in the infrastructure required to offer methadone maintenance services. While hospital inpatient programs already have the resources necessary to utilize medications, outpatient programs may be less equipped to do so. To examine whether adoption rates vary significantly by primary treatment modality, we control for whether the program *operates on an outpatient-only basis *(i.e., does not offer hospital inpatient or residential treatment services). At the time of the survey, the use of buprenorphine with adolescent clients had not been thoroughly studied; as a result, its adoption in adolescent treatment programs as of 2006 is expected to be lower than in programs serving predominantly adult clients. For the purpose of these analyses, N-SSATS facilities are classified as *adolescent programs *if at least 75% of their clients were under age 18 on the survey reference date.

The use of pharmacotherapies and other evidence-based practices are generally viewed as indicators of higher-quality treatment programs. Because accreditation is also a widely accepted indicator of program quality, it is controlled for in these analyses. Programs self-reported their *accreditation status *to N-SSATS, and a dichotomous variable is used. Analyses also include a measure of the *total number of admissions *to the treatment facility in the past year, with the expectation that larger facilities will have both the resources and customer base needed to support the adoption of buprenorphine.

Upon its approval by the FDA, buprenorphine was widely advertised as a viable option for the delivery of opioid dependence treatment in rural and remote areas, where patients lacked access to opioid treatment programs. To examine its early diffusion in this regard, we include a measure of *geographic location*. Specifically, N-SSATS indicates the metropolitan or micropolitan area (if any) where each facility is located. These have been reduced to a dichotomous variable, where 1 = located in metro/micropolitan area, and 0 = located outside these areas (i.e., rural counties).

Three measures of potential program funding sources are coded from the N-SSATS data file. The first is whether the program receives any *funds from federal, state, or local government sources*. Programs receiving funding from public sources are likely to be more influenced by state policies. Receipt of such funds is reported as a dichotomous variable in N-SSATS, and is coded as such in these analyses. A second N-SSATS variable indicates whether the treatment program *accepts Medicaid *as a form of payment for treatment services (1 = yes, 0 = no). A third funding-related variable indicates whether the program has any written arrangements with *managed care companies *(1 = yes, 0 = no). This is included as an indicator of reliance on private insurance, which is expected to be positively associated with buprenorphine adoption.

Finally, the dependent variable in these analyses is a single N-SSATS item indicating whether the program *uses buprenorphine *(1 = yes, 0 = no). In separate questions, facilities indicated whether they used Subutex and/or Suboxone. An affirmative response to either item resulted in a "yes" response on the buprenorphine adoption variable. N-SSATS does not provide sufficient detail to measure the extent of use within programs (e.g., the number of clients receiving the medication, or the frequency with which it is prescribed). In 2006, 11.4% of eligible N-SSATS respondents indicated that they prescribed buprenorphine. Table 1 provides descriptive statistics for each of these N-SSATS variables.

**Table 1:** Characteristics of N-SSATS facilities in analyses (N = 10,410)

Sample characteristics	Mean or Percent	Range (sd)
Ownership/Profit Status*		
Government owned	14.5%	0 – 1
Private, non-profit	61.0%	0 – 1
		
*Facility Characteristics*		
Based in a hospital	13.0%	0 – 1
Opioid Treatment Program (OTP)	8.8%	0 – 1
SA treatment is primary focus	61.3%	0 – 1
Offers detox services	21.8%	0 – 1
Outpatient only	61.3%	0 – 1
Adolescent program	6.6%	0 – 1
Accredited	43.7%	0 – 1
Past-year admissions	304.01	1 – 15000 (516.5)
Located in a metropolitan area	77.0%	0 – 1
		
*Key Funding Sources*		
Receives government funds	64.2%	0 – 1
Accepts Medicaid	55.1%	0 – 1
Has managed care contract(s)	50.8%	0 – 1
		
*Dependent variable*		
Uses buprenorphine	11.4%	0 – 1

*reference category = private for-profit

### Multivariate Models

Logistic regression models were estimated to test the effects of facility characteristics and state environments on the likelihood of adoption of buprenorphine by community-based treatment programs. Responses from the SSA/SMA interviews were attached to the record of each treatment program in the data set. As a result, programs within the same state have non-independent observations on the state environment variables; to account for this, the multivariate analyses were run using the survey ("svy") set of commands available in Stata v8.0, with state as the primary sampling unit. This approach produces robust standard errors and accounts for the effect of clustering and stratification in survey sample designs when calculating variance, standard errors, and confidence intervals [43].

Listwise deletion reduced the available N to 10,410 for the regression models. The majority of missing values were on the "past year admissions" variable. Facilities excluded from the analyses were not significantly different than those retained, either in their structural characteristics or in their adoption of buprenorphine (data not shown).

## Results

Estimates obtained in the multivariate logistic regression model are shown in Table 2. The ownership variables were significant predictors of buprenorphine adoption. Both government-owned facilities as well as non-profits were just over half as likely to have adopted buprenorphine relative to the reference category of private for-profit treatment programs. In terms of facility characteristics, both hospital-based programs and OTPs were twice as likely as other facilities to have adopted buprenorphine, net of the other variables in the model. Programs offering detoxification services were more than 7 times more likely than those without detox services to have adopted buprenorphine by 2006.

**Table 2:** Results of logistic regression of buprenorphine adoption on facility and state characteristics

	B	95% CI	OR
	
*Ownership/Profit Status*^*a *^			
Government owned	-.457*	(-.833, -.082)	.63
Private, non-profit	-.605**	(-.827, -.384)	.55
			
*Facility Characteristics*			
Based in a hospital	.671**	(.411, .931)	1.96
Opioid Treatment Program (OTP)	.696**	(.276, 1.115)	2.00
SA treatment is primary focus	.014	(-.332, .359)	
Offers detox	1.959**	(1.545, 2.374)	7.09
Outpatient only	-.186*	(-.343, -.028)	.83
Adolescent program	-1.123**	(-1.667, -.581)	.33
Accredited	.307**	(.114, .500)	1.36
Past-year admissions	.0002**	(.0001, .0003)	1.00
Located in a metropolitan area	.541**	(.243, .838)	1.72
			
*Key Funding Sources*			
Receives government funds	-.306**	(-.515, -.098)	.74
Accepts Medicaid	.175	(-.110, .460)	
Has managed care contract(s)	.472**	(.280, .665)	1.60
			
*State-Level Variables*			
SSA encourages use of buprenorphine	.171	(-.098, .440)	
Medicaid covers buprenorphine	.081*	(.011, .152)	1.09
McKelvey & Zavonia's R^2^	.364		
Model chisq (df) p	778.51 (16) p < .001		

N = 10,410. B = standardized coefficient; CI = confidence interval; OR = odds ratio (shown only for significant predictors).

^a^Reference category is for-profit treatment programs.

*p < .05, **p < .01

Several other structural characteristics were associated with the use of buprenorphine. As anticipated, programs serving predominantly adolescent clients were significantly less likely to have adopted buprenorphine compared to those serving adult populations. Accredited programs were about one-third more likely to use this medication, and it should be noted that this effect holds even with hospital status controlled. Larger facilities were also more likely to have adopted buprenorphine. While the coefficient for this variable appears trivial, this significant effect can be better appreciated by examining the effects of a standard deviation increase in caseload size. Specifically, each standard deviation increase in the number of past-year admissions was associated with a 10% increase in the odds of using buprenorphine. Net of the other variables, adoption of buprenorphine was about 72% more likely among programs located in metropolitan (versus rural) areas.

The financial variables showed mixed effects on the odds of adopting buprenorphine. Treatment programs that received any government funding were about 25% less likely than other programs to have adopted the medication. Conversely, programs having at least one managed care contract were 60% more likely than other programs to have been using buprenorphine in 2006. Whether a program accepted Medicaid payments was not predictive of buprenorphine adoption.

In terms of activity at the state level that was expected to affect programs' prescribing behaviors, there were mixed results. Programs located in states that were engaging in activities to encourage the use of buprenorphine were no more likely than programs in other states to have adopted buprenorphine in 2006. However, the availability of coverage for buprenorphine in state Medicaid plans had a significant effect on program-level adoption; the odds of programs offering buprenorphine were 8.5% greater in states where this benefit was available to patients. In all, the model explained about 36% of the variance in program-level adoption of buprenorphine.

## Discussion

These analyses are consistent with prior research indicating that a mix of structural, funding, and environmental variables are associated with organizational-level decisions to adopt pharmacotherapies. Structural variables available in N-SSATS suggest that the early adopters of buprenorphine were likely to be OTPs, along with facilities offering hospital-based, inpatient and detoxification services. These programs represent logical early inroads for buprenorphine. OTPs are challenged to meet the needs of opioid-dependent clients, and buprenorphine provides an alternative to methadone. Likewise, clinical trials showing buprenorphine to be effective for short-term detoxification may have contributed to its initial uptake among treatment programs offering detoxification services. Community-based treatment programs may view buprenorphine as a clinically effective and financially feasible alternative to either medically managed detoxification protocols or methadone maintenance options for their opioid-dependent clients. Finally, inpatient and hospital-based programs already have the medical infrastructure available to permit the use of pharmacotherapies, and buprenorphine likely represents an incremental addition to existing medication-based approaches in these settings, rather than a new line of services. As such, then, these programs are logical early adopters for buprenorphine and are probably least influenced by state policies and funding.

However, funding variables are also important in explaining variation in the current use of buprenorphine in community treatment programs. Government owned and government funded programs had significantly lower odds of adopting the medication, whereas private for-profit programs were most likely to offer buprenorphine. While acceptance of Medicaid as a payment source was not associated with the use of buprenorphine, coverage of the medication by Medicaid was a significant predictor. In this respect, states that were proactive in modifying the Medicaid formulary to include buprenorphine appear to have had a significant influence on program-level decisions to make this service available to their clients.

Net of the other variables measured, however, a state substance abuse agency's general orientation toward buprenorphine does not appear to have a measurable effect on program-level behavior. States that actively encouraged the use of buprenorphine (by distributing clinical guidelines, encouraging clients to be notified about the availability of the medication, and taking a supportive stance toward the medication) were no more likely to influence program decisions to adopt the medication than states with a less supportive approach. These results seem to suggest that the tangible signs of support for buprenorphine are most associated with the inclusion of the drug on the Medicaid formulary. Without a reliable source of payment for a measureable subset of clients entering treatment, programs are constrained in their choices of which services to offer. If clients will not be able to afford the medication, programs are unlikely to adopt it, regardless of the strength of the evidence base to support its use or the degree of encouragement from external stakeholders.

## Limitations

This study has several limitations that should be acknowledged. In terms of our primary data collection, we looked only at the actions and perspectives of the Single State Agency or the State Methadone Authority regarding buprenorphine, but not the activities of other related agencies such as the state Medicaid office or state pharmacy boards. Examining the activities of these partner agencies as well as regional or county agencies or professional associations within states was beyond the scope and resources of the study, but merits attention in future research.

N-SSATS is useful for obtaining a general overview of the state of the nation's treatment system, but the dataset necessarily trades depth for breadth. There are few predictor variables available to permit a meaningful understanding of the organizational factors influencing the adoption of evidence-based practices among treatment providers. Relevant predictors of buprenorphine adoption might include, for example, the number of physicians on staff, the percentage of clients with a primary opioid dependence diagnosis, and the percentage of clients with Medicaid as their primary expected form of payment. These measures are not available in N-SSATS. In particular, the lack of detail on client payment sources may have been partially responsible for the non-significant coefficient for the "accepts Medicaid" variable in these analyses. If a program's decision to adopt a new pharmacotherapy is influenced by clients' ability to pay, and if Medicaid is an indicator of this, then the relevant predictor variable is not whether the program accepts Medicaid, but the proportion of clients who are dependent on Medicaid for payment. Inferring the likely importance of Medicaid for populations served by public sector programs is not a substantial leap, but the N-SSATS data do not provide the opportunity to explore this association in detail.

Finally, it should be noted that state policies and clinical practice are both constantly changing. The findings presented in this article are a snapshot of an evolving process as it appeared in 2006, and may not reflect current policy or practice.

## Conclusion

The adoption of pharmacotherapies in substance abuse treatment has been relatively limited, despite the recent approval of several new medications for alcohol and opioid dependence. While a philosophical resistance to the medicalization of addictive disorders may undergird much of the slow diffusion, there are nevertheless identifiable structural impediments to the use of pharmacotherapies for addiction treatment. To the extent that these barriers can be reduced by targeted policy changes, patients' options for effective treatment services can be greatly increased.

These findings provide empirical support for the common perception that public sector clients are at a relative disadvantage in terms of access to state-of-the-art treatment. These analyses, based on data from more than 10,000 treatment programs across the U.S. in 2006, show that public sector, government funded programs are significantly less likely to have adopted buprenorphine for the treatment of opioid dependence. Programs in the for-profit sector, and those with privately-insured clients, are more likely to have adopted this medication. States that have incorporated buprenorphine into the Medicaid formulary have made inroads into closing this gap. It remains to be seen what additional actions are needed to further reduce these apparent disparities, as well as to identify whether and how state-level activities have influenced the adoption of other evidence-based treatment options.

Based on these analyses, as well as corroborating anecdotes from the telephone interviews conducted with SSA/SMA staff, it appears that most state oversight agencies regarded the Medicaid formulary as the end-point for state involvement in the diffusion of buprenorphine. While our respondents indicated no discernible resistance to the diffusion of buprenorphine in community-based treatment programs, there was also little in the way of observable behavior to actively promote its use, beyond support for clinician training or educational opportunities.

Buprenorphine faces neither the regulatory challenges associated with methadone, nor the barriers to diffusion that limited the uptake of LAAM. While a sizeable percentage of treatment programs (11.4%) had begun using it within the first three years of its availability, it remains to be seen how much this new medication will continue to penetrate the treatment system. Moreover, additional barriers to the use of buprenorphine remain to be identified at the client, clinician, program, and community levels. More detailed analyses that compare the predictors of adoption across the public and private sectors may yield important insights, particularly if variables other than insurance coverage show strong predictive effects in the private sector, where the use of medications is considerably more common [16,18]. An essential next step is to determine which, if any, of those barriers are amenable to change via revision of state or local policies or financing processes, and then identify and disseminate effective systems change solutions.

## Competing interests

The authors declare that they have no competing interests.

## Authors' contributions

LJD directed the research project and drafted the manuscript, AJA performed the data analysis and contributed to the methods and results sections. Both authors read and approved the final manuscript.
